# Deep hashing for global registration of untracked 2D laparoscopic ultrasound to CT

**DOI:** 10.1007/s11548-022-02605-3

**Published:** 2022-04-02

**Authors:** João Ramalhinho, Bongjin Koo, Nina Montaña-Brown, Shaheer U. Saeed, Ester Bonmati, Kurinchi Gurusamy, Stephen P. Pereira, Brian Davidson, Yipeng Hu, Matthew J. Clarkson

**Affiliations:** 1 Wellcome/EPSRC Centre for Interventional and Surgical Sciences and Centre for Medical Image Computing, UCL, London, UK; 2 Division of Surgery and Interventional Science, UCL, London, UK; 3 Institute for Liver and Digestive Health, UCL, London, UK

**Keywords:** Laparoscopic ultrasound, Multi-modal registration, Convolutional neural networks, Deep hashing

## Abstract

**Purpose:**

The registration of Laparoscopic Ultrasound (LUS) to CT can enhance the safety of laparoscopic liver surgery by providing the surgeon with awareness on the relative positioning between critical vessels and a tumour. In an effort to provide a translatable solution for this poorly constrained problem, Content-based Image Retrieval (CBIR) based on vessel information has been suggested as a method for obtaining a global coarse registration without using tracking information. However, the performance of these frameworks is limited by the use of non-generalisable handcrafted vessel features.

**Methods:**

We propose the use of a Deep Hashing (DH) network to directly convert vessel images from both LUS and CT into fixed size hash codes. During training, these codes are learnt from a patient-specific CT scan by supplying the network with triplets of vessel images which include both a registered and a mis-registered pair. Once hash codes have been learnt, they can be used to perform registration with CBIR methods.

**Results:**

We test a CBIR pipeline on 11 sequences of untracked LUS distributed across 5 clinical cases. Compared to a handcrafted feature approach, our model improves the registration success rate significantly from 48% to 61%, considering a 20 mm error as the threshold for a successful coarse registration.

**Conclusions:**

We present the first DH framework for interventional multi-modal registration tasks. The presented approach is easily generalisable to other registration problems, does not require annotated data for training, and may promote the translation of these techniques.

**Supplementary Information:**

The online version contains supplementary material available at 10.1007/s11548-022-02605-3.

## Introduction

Laparoscopic Ultrasound (LUS) is a frequently used imaging tool that allows surgeons to inspect critical blood vessels during laparoscopic liver resection [1]. Since LUS images have a small field of view and do not always clearly display the target tumour outline, their registration to a pre-operative CT scan has been suggested as an image-guidance method [2]. The main challenge in this registration problem is the size difference between LUS and CT - it is inherently difficult to match a small set of 2D vessels to a much larger 3D vessel tree. Due to this limitation, the majority of solutions present in the literature usually requires either a manual initialisation to the registration [3] or electromagnetic (EM) tracking to compound 3D LUS volumes [4, 5]. These two conditions currently prevent the translation of these guidance techniques. Therefore, we have previously proposed a novel Content-based Image Retrieval (CBIR) framework that is potentially capable of globally registering untracked 2D LUS images to CT [6]. However, this approach encodes vessel images with a limited set of handcrafted features that do not capture the vessel lumen shape variability, potentially compromising the overall registration performance. In this work, we propose the use of Convolutional Neural Networks (CNN) in a Deep Hashing (DH) framework to learn a more descriptive representation of vessel images and therefore increase the performance of the CBIR pipeline.

### Background

Few authors have tackled the specific registration of LUS to CT images of the liver, mainly due to the small size of LUS images and lack of 3D LUS probes [5]. The majority of approaches consider point-based registration algorithms using vessel features and were validated on phantoms [4, 7, 8] and *in vivo* [3, 5] animal data. However, all these methods require either a manual initialisation to the registration that is difficult to achieve during surgery, or EM tracking that is costly and not readily available for clinical LUS probes.

As a means of improving clinical translation, we have previously proposed the use of CBIR to obtain a global coarse registration without using tracking [6]. In our framework, instead of optimising an alignment, a database of possible registration solutions is pre-simulated from CT, and a vessel content feature vector is associated with each of them. For any input LUS images, vessel features are extracted, and registration is achieved by finding the best matching vectors in the database. In order for this approach to be computationally feasible, each vessel in a 2D image was simply defined by its centroid location and area. This encoding has two disadvantages: firstly, the centroid and area description do not uniquely describe large transversal vessel shapes; secondly, the size of feature vectors is defined by the number of vessel occurrences in an image, making the registration task sensitive to the number of segmented vessels in LUS.

An appealing solution to these problems is to use DH networks [9], where CNNs are used to directly extract image features and compress them to fixed size hash codes. Generally, hashing methods aim to map similar images to hash codes that are close in hashing space. Typically, similarity is defined by semantic labels, and DH methods learn these representations by training a network for an auxiliary task such as classification and extracting feature vectors directly from the fully connected layers that precede the output [10]. For registration, we are not focusing on any semantic meaning but on whether an image is aligned or not. Therefore, it is more sensible to learn codes from registered (similar) and mis-registered image pairs (dissimilar) using a Siamese Network (SN). Inspired by the SN hashing works in [11, 12], we propose the use of a triplet hashing framework to encode segmented vessel images. During training, we input image triplets from one segmented CT scan consisting of a vessel image, an accurately registered image and an inaccurately registered image. A contrastive loss is then used to minimise the distance between similar codes and maximise otherwise. Instead of learning through classification, we use an Autoencoder (AE) architecture for the self-reconstruction of the images and learn hash codes as their latent representation.

Our main contributions are:

1. A novel triplet training scheme that is suitable for the application of DH in multi-modal registration;
2. A self-supervised framework that does not require annotated data and can be generalised to other registration problems other than LUS to CT.

## Methods

The aim of our CBIR framework is to coarsely register a set of 2D untracked LUS images acquired continuously in time to a pre-operative CT volume and comprises two steps: an image retrieval task to find a pool of possible registration solutions for each LUS image, and an optimisation scheme to estimate the most likely sequence to represent the LUS acquisition. In this section, we focus mostly on the first step, where the proposed DH framework is integrated.

**Fig. 1:**
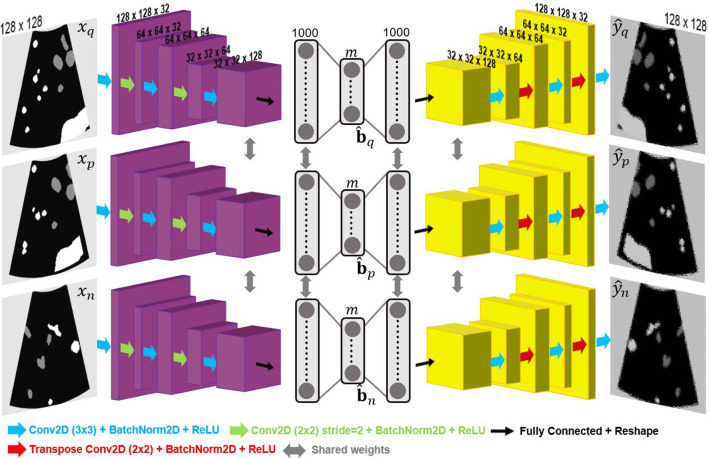
Proposed Siamese DH model based on an AE architecture. Coloured arrows represent convolutional operations. Grey blocks in the middle represent fully connected layers. Encoder and Decoder paths are highlighted in purple and yellow, respectively. During training, image triplets (${\mathrm {x}}_q$, ${\mathrm {x}}_p$, ${\mathrm {x}}_n$) are input to the model, and outputs are the respective hash code estimates (${\hat{\mathbf {b}}}_q$, ${\hat{\mathbf {b}}}_p$, ${\hat{\mathbf {b}}}_n$) and decoded reconstructions (${\hat{\mathrm {y}}}_q$, ${\hat{\mathrm {y}}}_p$, $\hat{\mathrm {y}}_n$)

### Deep hashing framework

We propose the use of a SN that follows a 2D encoder-decoder architecture and has three branches, as illustrated in Fig. 1. During training, the network receives three segmented vessel image inputs: ${\mathrm {x}}_{q}$, the query image which represents the image to which we intend to retrieve a registration solution; ${\mathrm {x}}_{p}$, the positive image, which is “relevant” to the query and given by an accurate registration to ${\mathrm {x}}_{q}$, and finally ${\mathrm {x}}_{n}$, the negative image which is not “relevant” to the query and given by a mis-registration of ${\mathrm {x}}_{q}$.

To learn image features for hashing, we first pass the three images separately through three encoders with shared weights (purple blocks in Fig. 1) composed of five convolutional layers that are each followed by batch normalisation and activated with ReLU. Alternately, we increase the number of channels with 3$\times$3 convolutions (blue arrows) and decrease feature map dimensions with 2$\times$2 convolutions (green arrows) with a stride of 2. Hash codes $\hat{\mathbf {b}}_{q}$, $\hat{\mathbf {b}}_{p}$ and $\hat{\mathbf {b}}_{n}$ $\in \mathbb R^m, m=32$ are estimated after passing the resulting features through two fully connected layers, and the two following losses are computed:

1 $$\begin{aligned} {\mathcal {L}}_{1}&= {\text {max}}\{r \cdot m - \Vert {\hat{\mathbf {b}}}_{q} - {\hat{\mathbf {b}}}_{n}\Vert ^2 + \Vert {\hat{\mathbf {b}}}_{q} - {\hat{\mathbf {b}}}_{p}\Vert ^2, 0\} \end{aligned}$$

2 $$\begin{aligned} {\mathcal {L}}_{2}&= \Vert |{\hat{\mathbf {b}}}_{q}| - {\mathbf {1}}\Vert ^2 + \Vert |{\hat{\mathbf {b}}}_{p}| - {\mathbf {1}}\Vert ^2 + \Vert |{\hat{\mathbf {b}}}_{n}| - {\mathbf {1}}\Vert ^2. \end{aligned}$$

${\mathcal {L}}_{1}$ is the triplet contrastive loss presented in [12] that minimises the $L^2$ norm between ${\hat{\mathbf {b}}}_{q}$ and ${\hat{\mathbf {b}}}_{p}$ and maximises between ${\hat{\mathbf {b}}}_{q}$ and ${\hat{\mathbf {b}}}_{n}$. To prevent the loss from being dominated by the negative pairing difference, we do not allow the distance between the negative and query codes to contribute with more than a limit of *r* times the length of the codes. ${\mathcal {L}}_{2}$ is the binarisation loss presented in [11], that enforces code values to approximate either 1 or -1, and not diverge while also minimising ${\mathcal {L}}_{1}$. This loss is measured separately for each code, and ${\mathbf {1}}$ represents a vector with the hash code length filled with 1.

After the encoding path forward pass, resultant codes are provided to the decoding path of the network (yellow blocks in Fig. 1), which has a symmetrical architecture with two fully connected and five convolutional layers, and uses 2$\times$2 transpose convolutions for image upsampling. The decoder predictions ${\mathrm {y}}_{q}$, ${\mathrm {y}}_{p}$, ${\mathrm {y}}_{n}$ are then used to calculate a reconstruction loss,

3 $$\begin{aligned} {\mathcal {L}}_{3}&= \sum {(\Vert {\mathrm {x}}_{q}-{\hat{\mathrm {y}}}_{q}\Vert ^2} + \Vert \mathrm {x}_{p}-{\hat{\mathrm {y}}}_{p}\Vert ^2 + \Vert \mathrm {x}_{n}-{\hat{\mathrm {y}}}_{n}\Vert ^2) \times 1/s \end{aligned}$$

where *s* is the image size. The final triplet loss is then given by

4 $$\begin{aligned} {\mathcal {L}}&= w_{1}{\mathcal {L}}_1 + w_{2}{\mathcal {L}}_2 + w_3{\mathcal {L}}_3 \end{aligned}$$

where $w_1$, $w_2$ and $w_3$ are predefined hyper-parameters.

#### Training strategy

To train the model of Fig. 1, we simulate 2D vessel images with the geometry of LUS from a patient-specific segmented CT volume. We create a fixed dataset of 2D images by pre-defining a set of virtual probe poses across the CT liver surface. Following the formulation presented in [6], each pose is defined by a position in the liver surface, a depth translation *d* across the inner liver surface normal and three rotations $R_{x}$, $R_{y}$, $R_{z}$ from a predefined rotation reference. This reference is defined by placing the probe shaft orthogonal to the liver surface and aligning the imaging plane with the sagittal plane. The dataset is then defined by a number of positions and a set of rotation and depth ranges.

To sample triplets from the dataset, we define $\mathrm {x}_q$ as a random image, $\mathrm {x}_p$ as a modified version of $\mathrm {x}_q$, and $\mathrm {x}_n$ as another random image whose pose is different from that of $\mathrm {x}_q$ (an example is displayed in Fig. 1). Intuitively, the pair of $\mathrm {x}_q$ and $\mathrm {x}_p$ is supposed to mimic the LUS to CT registration, where deformation and missing vessel sections are frequent. Therefore, $\mathrm {x}_p$ is obtained from $\mathrm {x}_q$ after applying uniform random displacements with a maximum amplitude of $d_p$ (deformation), and removing vessel sections up to a ratio of $m_p$ (missing data). For $\mathrm {x}_n$, we set thresholds for the minimum pose rotation and translation errors towards $\mathrm {x}_q$, $\theta _n$ and $t_n$.

Resulting vessel maps are input in the network as binary images. To integrate vessel label information from the portal vein and hepatic vein as in [6], we assign different integer values (1 and 2) to each of the two labels.

### Registration

Once the DH model is trained, registrations are performed by employing the previous CBIR framework [6]. In summary, a large set of images is simulated from CT and encoded with our DH model to generate a database of hash codes and associated registration solutions; for a set of typically *N*=3 LUS images, the same model is used to extract hash codes; retrieval is performed by comparing each LUS hash code to the database and finding *k* closest matches in a lowest Euclidean distance sense; the resulting retrieved poses are combined in a discrete Hidden Markov Model (HMM) to estimate the most kinematically likely sequence of CT images to represent the LUS acquisition.

**Table 1:** Data description of untracked LUS data used for retrieval and registration per clinical case. Sweeps refers to the number of continuous LUS acquisitions and Images to the number of images processed in each sequence. GT Images refers to the number of images in each sequence for which matching LUS and CT landmarks are available

Cases	1			2		3		4	5			Total
#Sweeps	3			2		2		1	3			**11**
#Images	58	24	51	24	8	62	66	44	49	28	48	**463**
#GT Images	3	4	5	5	2	7	9	7	10	5	6	**63**

## Experiments

### Data description

We validate our DH model on retrospective LUS and CT data from 5 clinical cases as in [6]. A total of 11 continuous sweeps of LUS images with dimensions 544$\times$664 and resolution 0.12 mm$\times$0.12 mm were acquired from the right lobe of the liver at a frequency of 40 Hz with a BK Medical 4 Way I12C4f probe^1^ using the NifTK software platform [13]. LUS vessels were manually segmented and labelled as portal vein or hepatic vein. A description of the number LUS images processed per sweep is presented in Table 1. Liver surface, hepatic and portal vein models were extracted from CT using a commercial service.^2^

### Model training

We train our model for 15 epochs on an NVIDIA GeForce RTX 3070 8GB graphics card using the Adam optimiser with a learning rate of $10^{-4}$, a batch size of 24 and data from a CT scan from one of the 5 cases. We specifically pick a CT where vessels were more finely segmented to provide the network with a wider distribution of vessel configurations. We slice ultrasound shaped planes from CT using an open source package [14] developed on top of the PythonTemplate from Scikit-Surgery [15]. Pose ranges and total number of images in the training dataset are listed in the left side of Table 2. Input vessel image triplets are resized to $128\times 128$. For the positive pairs, we set the maximum deformation $d_p = 5\,\hbox {mm}$ and the missing ratio $m_p = 0.25$. For the negative pairs, we use minimum errors $t_n = 20~\hbox {mm}$ and $\theta _n = 40^{\circ }$. We consider an $r = 0.5$ in the contrastive loss $\mathcal {L}_1$ [12] and loss weights of $w_1 = 10$, $w_2 = 1$, $w_3 = 100$ in $\mathcal {L}$. Weights were adjusted using a small subset of the training dataset so that each loss term contributed equally to the training. We train models with single and multiple labels separately.

**Table 2:** Pose range and resolution parameters for hashing databases. “Training” shows parameters used for training, whereas “LUS to CT Tests” shows parameters for testing. Rotations $R_x$, $R_y$, $R_z$ and depth *d* are presented with lower bound, upper bound and resolution step. Points represents the number of sampled surface positions, and Total the overall number of samples (k stands for thousand and M for million)

Cases	Training			LUS to CT Tests				
	3			1	2	3	4	5
$R_x (^{\circ })$	[0,	45,	45]		$[-40,$	40,	10]	
$R_y (^{\circ })$	$[-90,$	90,	45]		$[-90,$	0,	10]	
$R_z (^{\circ })$	[0,	45,	45]		$[-40,$	40,	10]	
*d*(*mm*)	[10,	20,	10]		[10,	25,	5]	
#Points	5073			3298	4637	3643	3467	3324
Total	203k			10.7M	15.0M	11.8M	11.2M	10.8M

### LUS to CT registration tests

To evaluate the performance of the whole LUS to CT registration pipeline, we test retrieval and registration separately for both our new DH method and the previous handcrafted feature-based CBIR method [6]. For both tests and models, we infer databases for each clinical case according to the pose ranges listed in the right section of Table 2. For Case 3, a small rotation perturbation is applied to the probe reference rotation so that training and testing consider always different images. We define a non-symmetrical range for $R_{y}$ since the LUS probe access to the right lobe of the liver constrains the probe swabbing across the surface mainly to one side.

#### Retrieval

Similarly to the experiment reported in [6], we test retrieval on a set of 63 LUS images distributed across the 11 sweeps (last row of Table 1). For each of these LUS images, we retrieve a set of *k*=200 candidate poses from the respective CT database, and measure retrieval precision for registration. Given the pool of retrieved poses, we define precision as the ratio of poses that are relevant to the input LUS image—in this case, relevance is defined by a maximum LUS to CT registration error threshold of 20 mm. We consider this value as sufficient for a global registration initialisation that can be further refined. As described in [6], we use the Target Registration Error (TRE) on a set of landmarks common in each of the LUS images and 3D CT volume. Additionally, we report the relevance rank, defined as the ordered index of the first closest database hash that results in a pose with TRE below 20 mm.

#### Registration

For the registration tests, we register the 11 sweeps using the discrete HMM optimisation detailed in [6] and the results of the experiments. Briefly, this HMM estimates the sequence of candidate poses in CT that best represents the LUS acquisition in time, assuming that images close in time should be also close in pose parameters. Compared to previous work, instead of attempting to register the complete sweep with a single optimisation, we register each LUS image in a sweep separately by simply considering only the previous LUS image candidate poses. In the formulation in [6], this means that we consider a graphical model with height *k*=200 and width *N*=2 for the registration of every image in the LUS sweep. This “window” optimisation does not allow the HMM to consider all images in the LUS sweep at once and return an inaccurate registration in case the retrieval task fails to retrieve an accurate pose in at least one LUS image.

For each sequence, we measure the success rate as the ratio of images for which the final registration error is below 20 mm. Since we do not have matching landmarks for all images, in this experiment we consider as error the Root Mean Square (RMS) of the distance between the sampled LUS image plane points of the obtained registration and a ground truth alignment. To obtain ground truth alignments for complete sequences, we first use a point-based registration [16] to align each of the LUS images for which landmarks are available to CT (listed in the last row of Table 1 and tested in the retrieval experiment). Using the resulting point-based registered poses as anchors, we interpolate translation and rotation parameters in time to generate approximate ground truth solutions for the remaining sweep images.

**Fig. 2:**
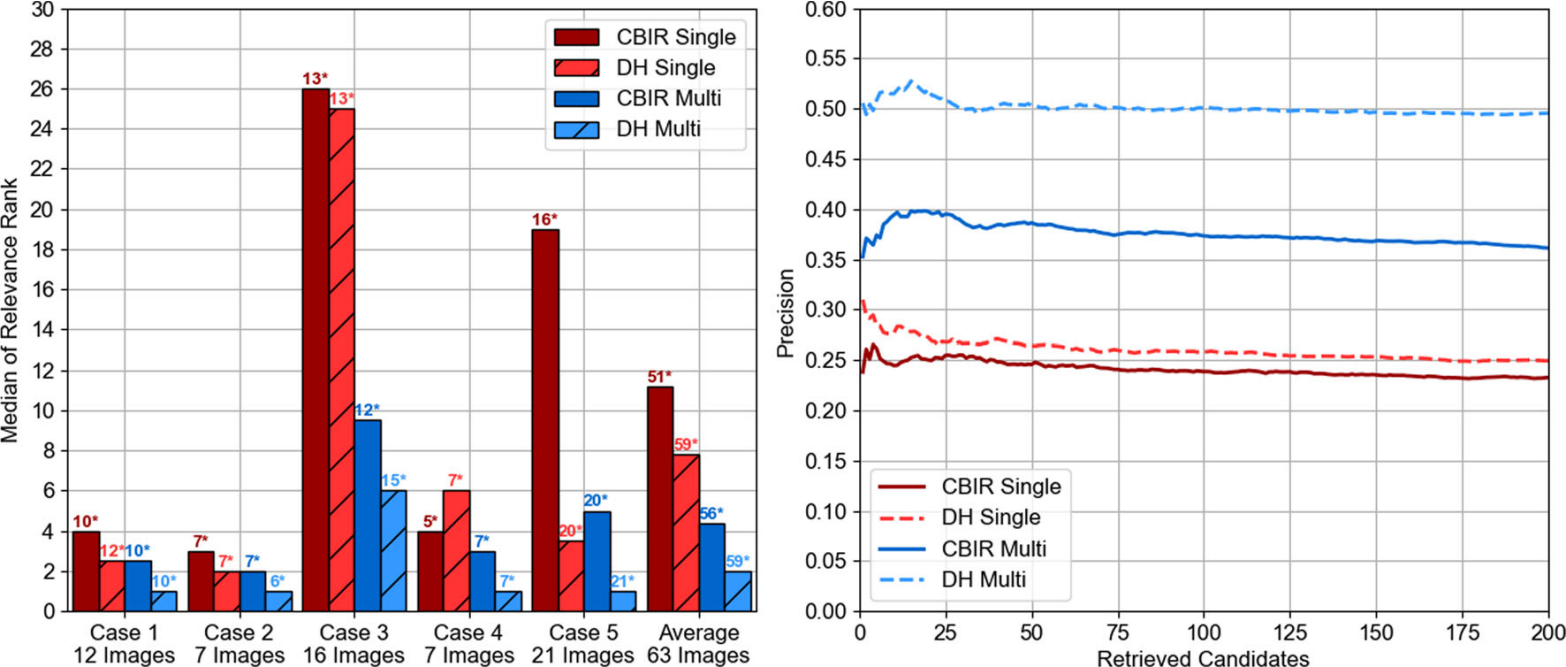
Retrieval performance across handcrafted CBIR and DH methods on 63 LUS images from 5 patient cases using *k*=200 candidates and considering a TRE below 20 mm as retrieval relevance criterion. Left shows the median of the rank at which a relevant image is retrieved. Above each bar, a number followed by * represents the number of images for which at least one relevant solution was retrieved. The number of images tested per case is displayed in the horizontal axis. Right shows the case averaged retrieval precision versus number of retrieved candidates. Red colour represents single-labelled results, and blue represents multi-labelled

## Results

### Retrieval

Results for the retrieval experiments are displayed in Fig. 2. We consider results for four approaches, the handcrafted feature CBIR and the new DH model with both single and multi-labelled encodings.

In the left bar chart, we show the median relevance rank measured for each patient case. On average, the DH models show a decrease in the relevance rank when compared with handcrafted CBIR, suggesting that DH requires fewer retrieved candidates to find a sufficiently accurate registration solution. Without labels, the number of required candidates decreases from 11 to 7, whereas with labels it decreases from 4 to 2. As expected, relevance rank is lower when multiple labels are considered. On top of each bar, we also display the number of images for which the retrieval task returns at least one solution with TRE below 20 mm. These numbers also show that the DH models are more likely to retrieve a coarse registration with a fixed number of candidates.

In the right plot, we display curves with the retrieval precision for registration versus number of retrieved candidates. Each curve represents average results of all images over all patients. The highest precision values around 0.5 are observed for the DH model with vessel labels, suggesting that on average, for any retrieved number of candidate poses, half of them will result in a sub-20 mm TRE. Lower precision values are observed for both single label models, with DH showing a slight increase from values around 0.25 to 0.27. These results show that the inclusion of labels significantly increases the performance, regardless of the encoding model used. Also, the improvement of DH is more pronounced in the multi-labelled option.

**Fig. 3:**
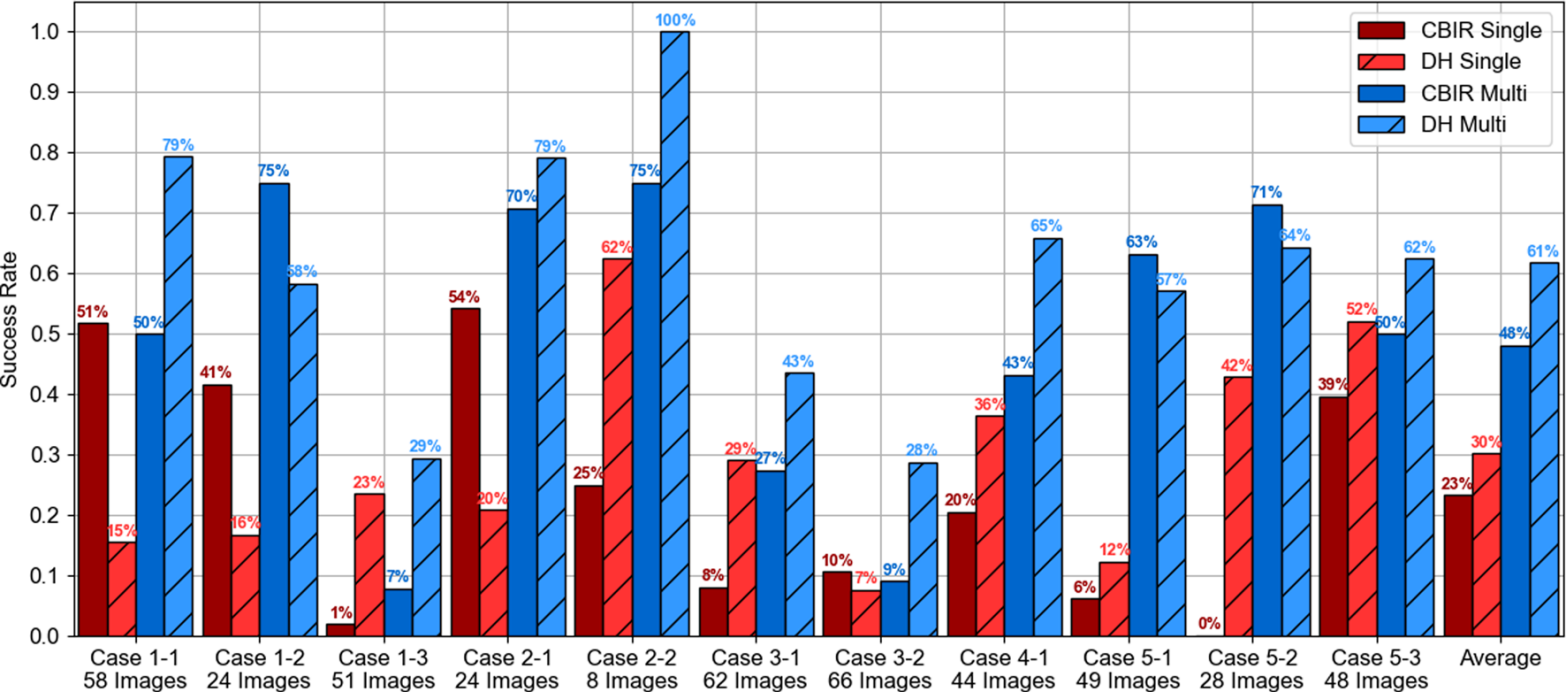
Registration success rate across 11 sweeps of untracked LUS from 5 patient cases considering a plane RMS error below 20 mm as success using DH and handcrafted CBIR methods. Red colour represents single-labelled results, and blue represents multi-labelled. For clarity, the success rate is displayed above each bar as a percentage

### Sweep registration

Sweep registration success rates are presented for the four methods in Fig. 3. Overall, the success rate follows the same trend as the retrieval tests—multi-labelled DH leads to the best performance with an average success rate of 61% and is followed by 48% obtained by the multi-labelled CBIR. Specifically, DH improves the success rate in 8 out of 11 sweeps over handcrafted CBIR and shows comparable performance in the remaining 3. These improvements are clearer in the sweeps of Case 3 and sweep 3 of Case 1, where the handcrafted methods perform very poorly. For the single label models, the DH improvement is not as clear, as sweeps 1 and 2 of case 1 perform clearly better with handcrafted features. Average registration accuracies were similar for all methods, ranging from 13.73 mm with multi-labelled DH to 15.47 mm with single-labelled CBIR. Detailed accuracy results are presented in supplementary material.

**Fig. 4:**
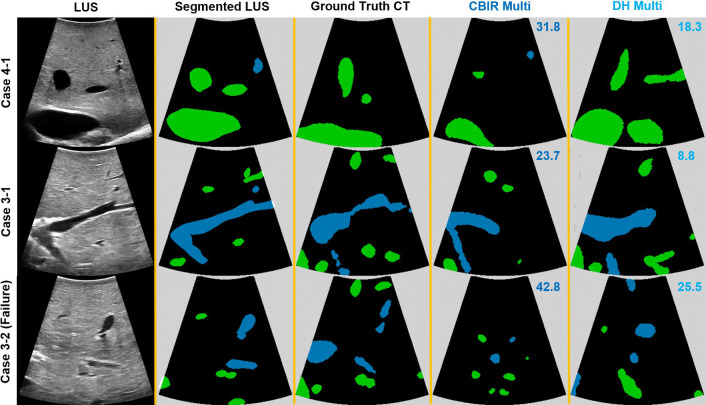
Examples of 2D registration results from 3 registered sweeps using multi-labelled DH and handcrafted CBIR. From left to right are shown the original LUS image, the LUS image segmentation and the 2D CT images obtained with the ground truth alignment, the handcrafted CBIR, and the DH model. For estimated solutions, we present the plane RMS error in the upper right corner of the respective CT image. Each row refers to a different LUS sweep. Green refers to hepatic vein and blue to portal vein. 3D visualisations of these results are included in the supplementary materials

Registration examples of one image from three distinct sweeps are displayed in Fig. 4. In these images, we can see in each row the LUS image and corresponding labelled segmentation, the matching ground truth 2D CT solution, and the 2D CT solutions obtained with both multi-labelled handcrafted CBIR and DH. In the first example of sweep 1 of case 4, we can see that compared to handcrafted CBIR, the DH approach finds a solution that better matches the large area of the vessel sections, and not just their position. A similar effect is observed in the second row with a large transversal section of the portal vein (blue)—DH performs a better shape matching in this case as well. In the third row, we see a registration example where both approaches fail. However, it is clear the handcrafted solution mostly makes use of vessel centroid positions for a matching.

## Discussion

Our results show that our novel DH approach shows substantial improvements over the previous handcrafted approach. As expected, the performance of multi-labelled methods greatly surpasses the single-labelled ones, as it reduces registration matching ambiguity. On average, the best registration and retrieval performances with a precision of 0.5 and registration success rate of 0.61 were obtained using DH with multi-labelled encoding.

In 3 out of 11 sweeps, the previous CBIR approach showed better results than DH. This may be explained by the fact that the dataset used to train the DH model did not contain the vessel shapes captured in these 3 sweeps, leading to worse performances. Despite these slightly poorer performances, the DH approach offers other computational improvements. Firstly, the retrieval task is computationally faster, as instead of comparing lists with different sizes and rejecting vessel section for each comparison [6], we can just directly compare fixed-size hash codes. Secondly, in the handcrafted approach we consider a range of lists to search according to the number of vessels present in the input. With DH, better results were obtained even when comparing the input without this refinement, highlighting the robustness of the approach to vessel topology. We also hypothesise that performance could be improved by including more variability in vessel images seen during training, by including images from multiple CT scans for example. Another advantage of this framework is generalisability. In the training process, a general feature encoding is learnt with automatically generated image examples irregardless of pose parameters. This means that the network does not learn patient-specific pose parameters and can be applied to other patients. From our understanding, the improvements observed in Case 3 are explained by the DH capability of encoding large vessels, and not the fact that the same CT volume was seen during training and testing.

The main limitation of our work is the rigidity assumption. Even though we demonstrate registration improvements, registering images such as the ones of Sweep 2 of Case 3 (Fig. 4) potentially requires modelling deformations induced by probe contact and abdominal insufflation. Future work will consider modelling these deformations in 2D images during simulation. We tested our method with manual segmentations, but there is undergoing work on automating this process [17, 18].

## Conclusions

We present the first DH approach for interventional multi-modal registration. This new deep learning framework shows improvements in the registration of LUS to CT over a classical CBIR approach. The framework is self-supervised and follows a Siamese triplet hashing scheme that does not require annotated data and can learn from few patient cases. This methodology could be potentially applied to other interventional registration problems such as Endoscopic Ultrasound to CT [19], given appropriate anatomical features.

## Supplementary Information

Below is the link to the electronic supplementary material.

Accuracy measurements and visual results description (127 KB)

DH result for Sweep 1 of Case 1 (4805 KB)

Handcrafted CBIR result for Sweep 1 of Case 1 (4798 KB)

DH result for Sweep 1 of Case 2 (2202 KB)

Handcrafted CBIR result for Sweep 1 of Case 2 (2169 KB)

DH result for Sweep 1 of Case 3 (5844 KB)

Handcrafted CBIR result for Sweep 1 of Case 3 (5841 KB)

DH result for Sweep 2 of Case 3 (6369 KB)

Handcrafted CBIR result for Sweep 2 of Case 3 (6306 KB)

DH result for Sweep 1 of Case 4 (3838 KB)

Handcrafted CBIR result for Sweep 1 of Case 4 (3814 KB)

## Footnotes

1 www.bkmedical.com.

2 www.visiblepatient.com.
