# Supplementary material for: Deep hashing for global registration of untracked 2D laparoscopic ultrasound to CT
Source: Int J Comput Assist Radiol Surg. 2022 Apr 2;17(8):1461–8. doi: 10.1007/s11548-022-02605-3 (PMC9307559; doi:10.1007/s11548-022-02605-3)
Supplement: Supplementary file 1 — Accuracy measurements and visual results description (127 KB) [file 11548_2022_2605_MOESM1_ESM.pdf]

Supplementary material of paper "*Deep hashing for global registration of untracked 2D laparoscopic ultrasound to CT*"

A – Registration Accuracy Results

	**Case 1**			**Case 2**		**Case 3**		**Case 4**	**Case 5**			**Average**
	1	2	3	1	2	1	2	1	1	2	3	
**CBIR Single**	17.42	13.85	19.10	13.23	8.91	18.44	16.49	16.87	14.80	-	15.53	**15.47**
**DH Single**	15.74	16.65	13.85	10.04	15.68	14.42	14.95	16.40	15.21	13.35	10.80	**14.49**
**CBIR Multi**	13.05	13.12	16.15	12.21	6.18	14.38	16.22	17.50	10.93	13.87	14.68	**13.85**
**DH Multi**	10.18	15.89	14.60	10.78	11.06	12.21	16.95	16.53	11.98	13.25	13.28	**13.73**

Supplementary Table 1. Average registration accuracy for 11 sweeps distributed across 5 tested clinical cases with both Classical CBIR and Deep Hashing methods.

B – Visual Registration Results

Visual registration results are presented as a .gif file per each LUS sweep. Each of these files show a sequence registration with either the Deep Hashing model (DH) or the handcrafted feature Content-based Image Retrieval (CBIR) method.

-> Three images are displayed at each time-point.

1. Top is Laparoscopic Ultrasound.
2. Middle is Ground Truth Registration 2D CT result.
3. Bottom is the obtained solution.

-> Two Laparoscopic Ultrasound shaped planes are overlayed in the CT at each time-point:

1. Black represents Ground truth.
2. Yellow represents obtained solution.

-> A string displays the RMS error between ground truth and obtained solution. Error message is green when error is below 20 mm.

Results for Fig.4 examples in the paper are as follows:

-> Top row can be found at Image time-point 7 in files 11548_2022_2605_MOESM10_ESM.gif (Deep Hashing) and 11548_2022_2605_MOESM11_ESM.gif (Handcrafted CBIR) which refer to Sweep 1 of Case 4.

-> Middle row can be found at Image time-point 17 in files 11548_2022_2605_MOESM6_ESM.gif (Deep Hashing) and 11548_2022_2605_MOESM7_ESM.gif (Handcrafted CBIR) which refer to Sweep 1 of Case 3.

-> Top row can be found at Image time-point 17 in files 11548_2022_2605_MOESM8_ESM.gif (Deep Hashing) and 11548_2022_2605_MOESM9_ESM.gif (Handcrafted CBIR) which refer to Sweep 2 of Case 3.
