# Supplementary figures and images for: Deep hashing for global registration of untracked 2D laparoscopic ultrasound to CT
Source: Int J Comput Assist Radiol Surg. 2022 Apr 2;17(8):1461–8. doi: 10.1007/s11548-022-02605-3 (PMC9307559; doi:10.1007/s11548-022-02605-3)

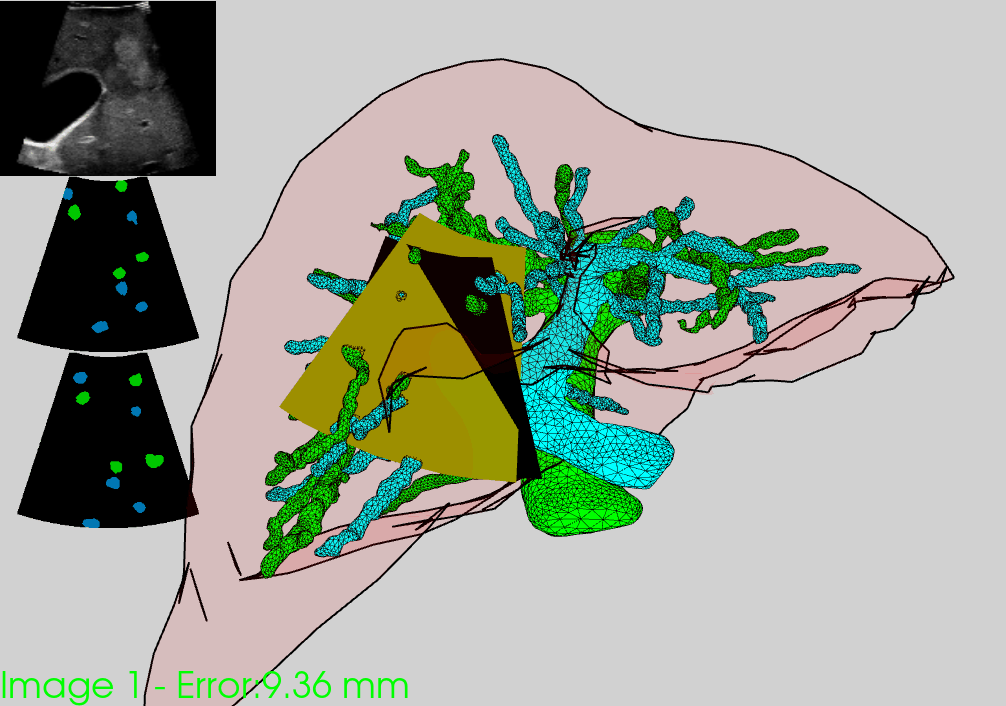

Supplement: Supplementary file 2 — DH result for Sweep 1 of Case 1 (4805 KB) [file 11548_2022_2605_MOESM2_ESM.gif]

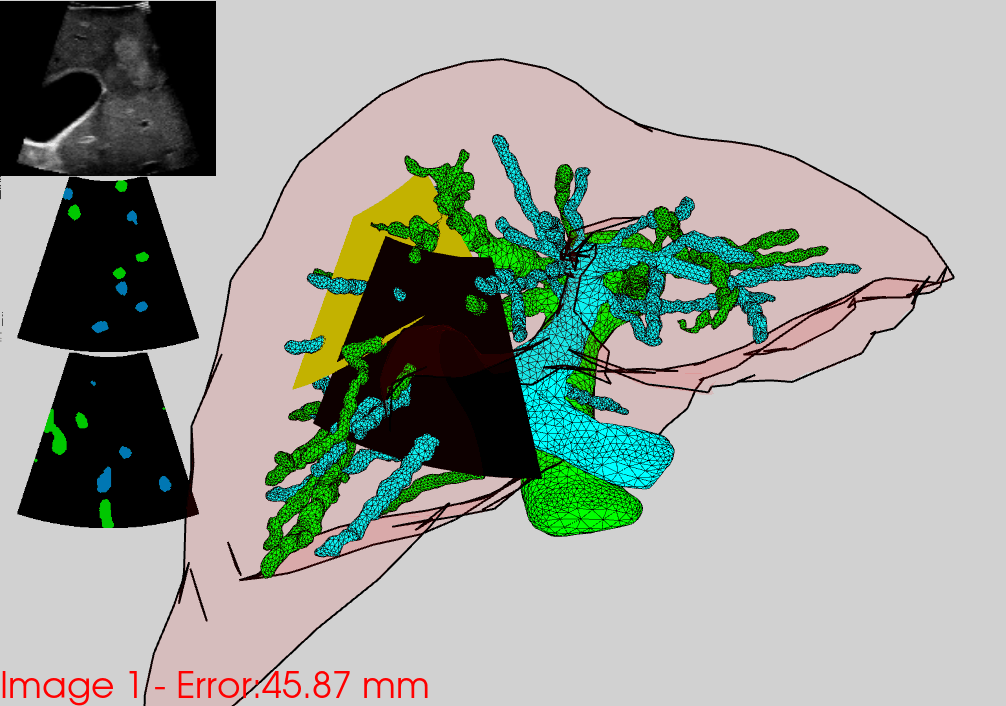

Supplement: Supplementary file 3 — Handcrafted CBIR result for Sweep 1 of Case 1 (4798 KB) [file 11548_2022_2605_MOESM3_ESM.gif]

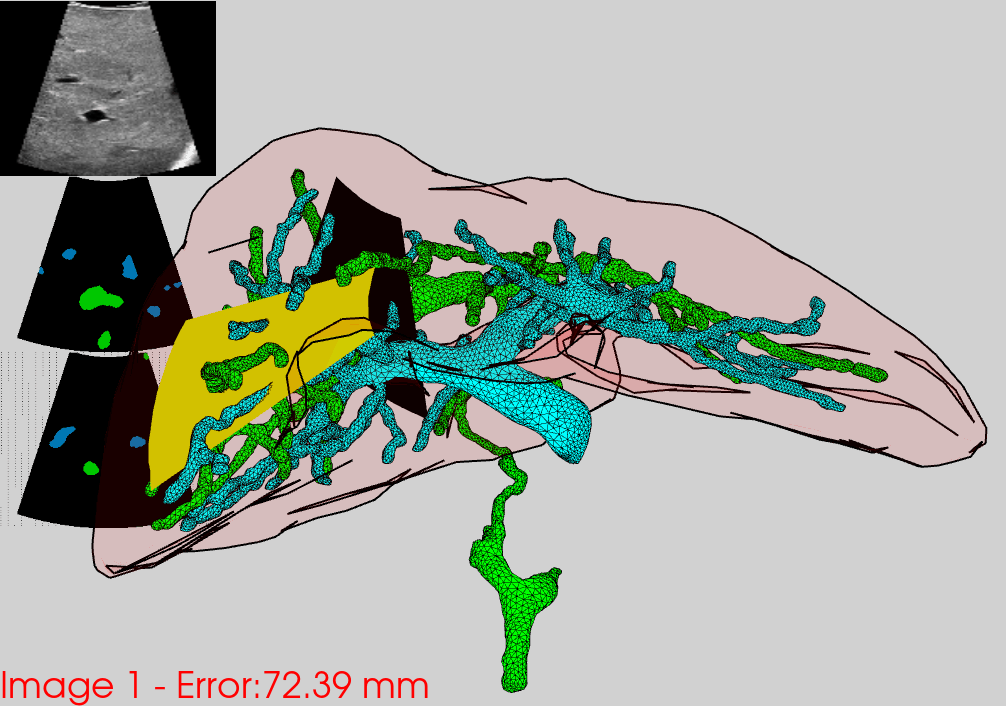

Supplement: Supplementary file 4 — DH result for Sweep 1 of Case 2 (2202 KB) [file 11548_2022_2605_MOESM4_ESM.gif]

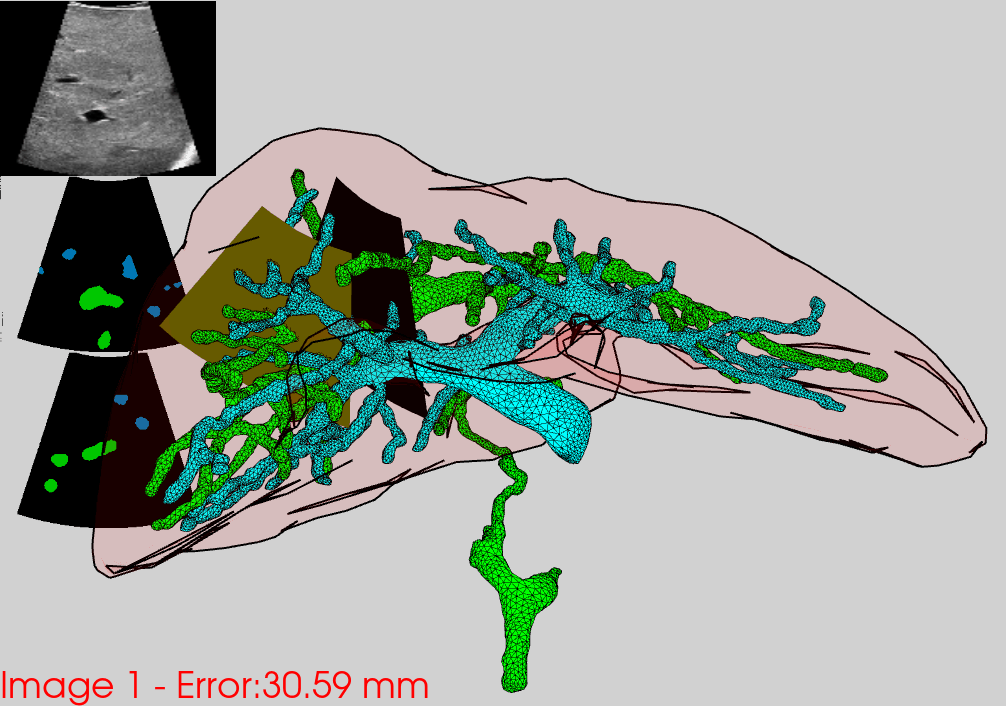

Supplement: Supplementary file 5 — Handcrafted CBIR result for Sweep 1 of Case 2 (2169 KB) [file 11548_2022_2605_MOESM5_ESM.gif]

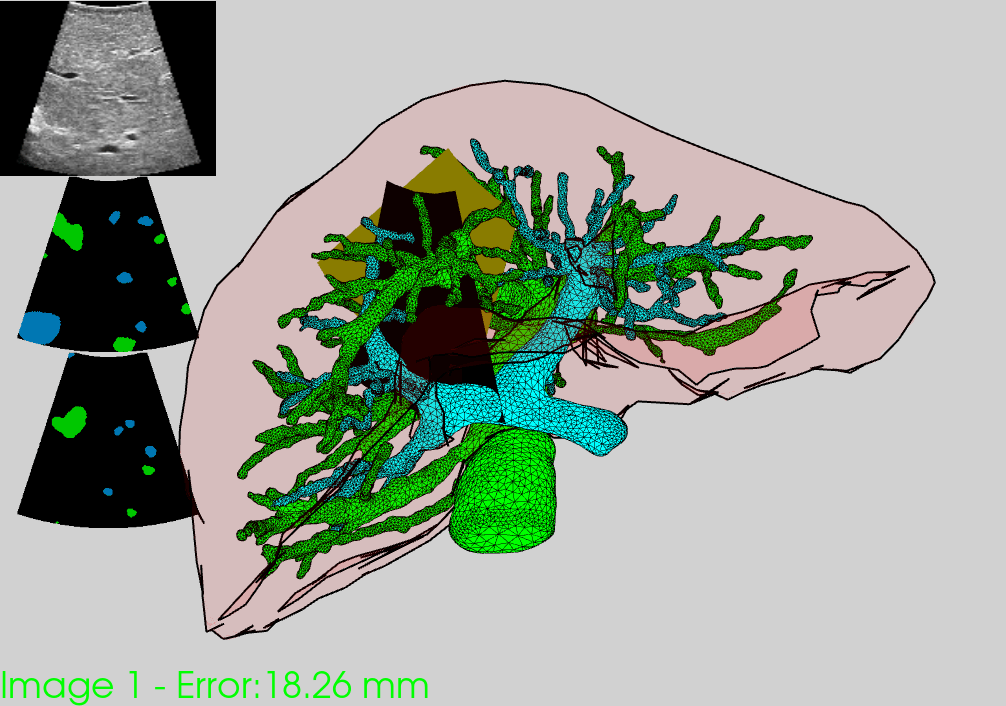

Supplement: Supplementary file 6 — DH result for Sweep 1 of Case 3 (5844 KB) [file 11548_2022_2605_MOESM6_ESM.gif]

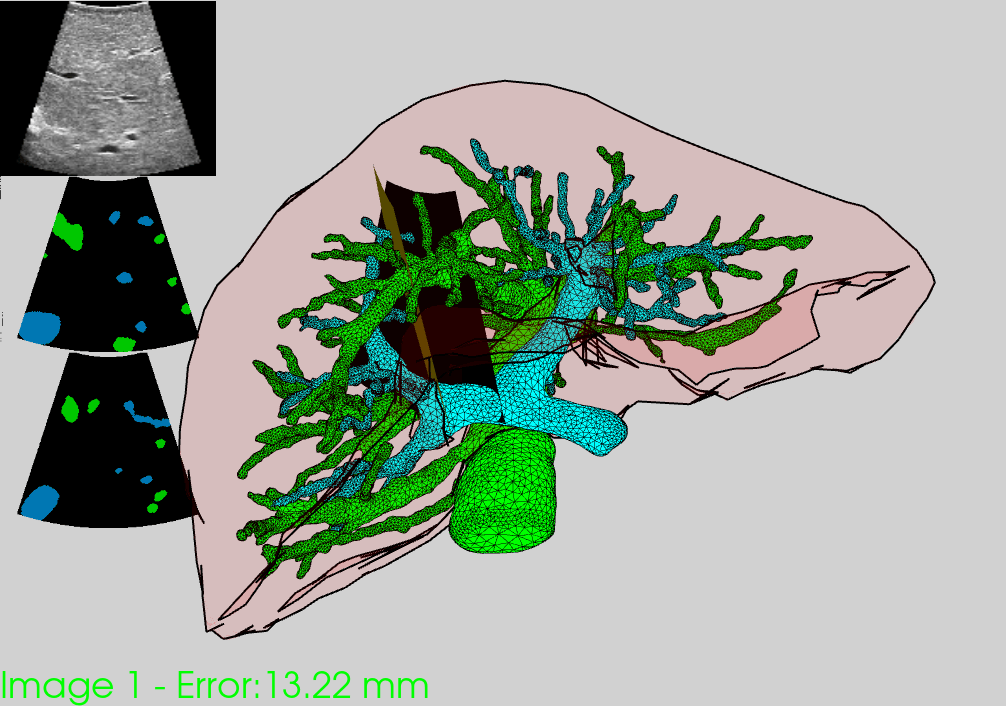

Supplement: Supplementary file 7 — Handcrafted CBIR result for Sweep 1 of Case 3 (5841 KB) [file 11548_2022_2605_MOESM7_ESM.gif]

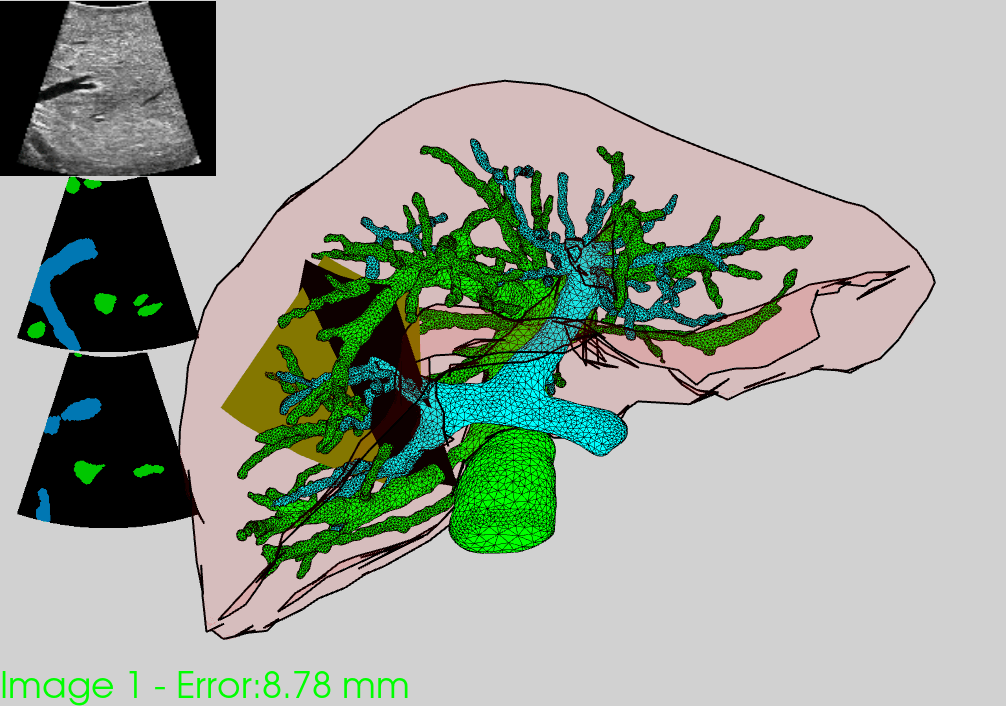

Supplement: Supplementary file 8 — DH result for Sweep 2 of Case 3 (6369 KB) [file 11548_2022_2605_MOESM8_ESM.gif]

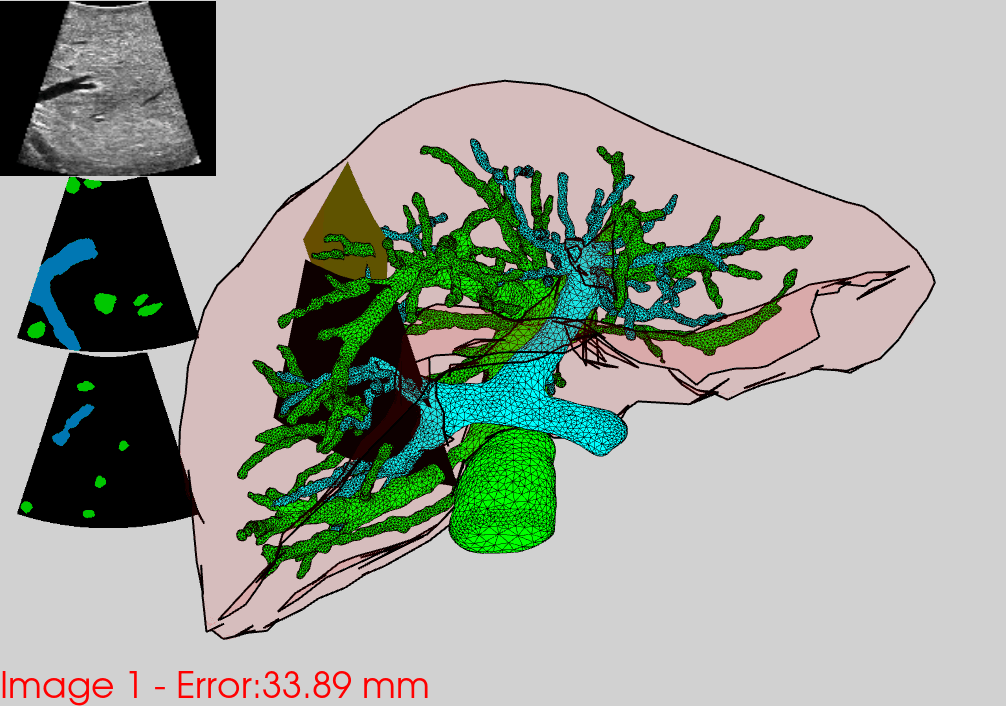

Supplement: Supplementary file 9 — Handcrafted CBIR result for Sweep 2 of Case 3 (6306 KB) [file 11548_2022_2605_MOESM9_ESM.gif]

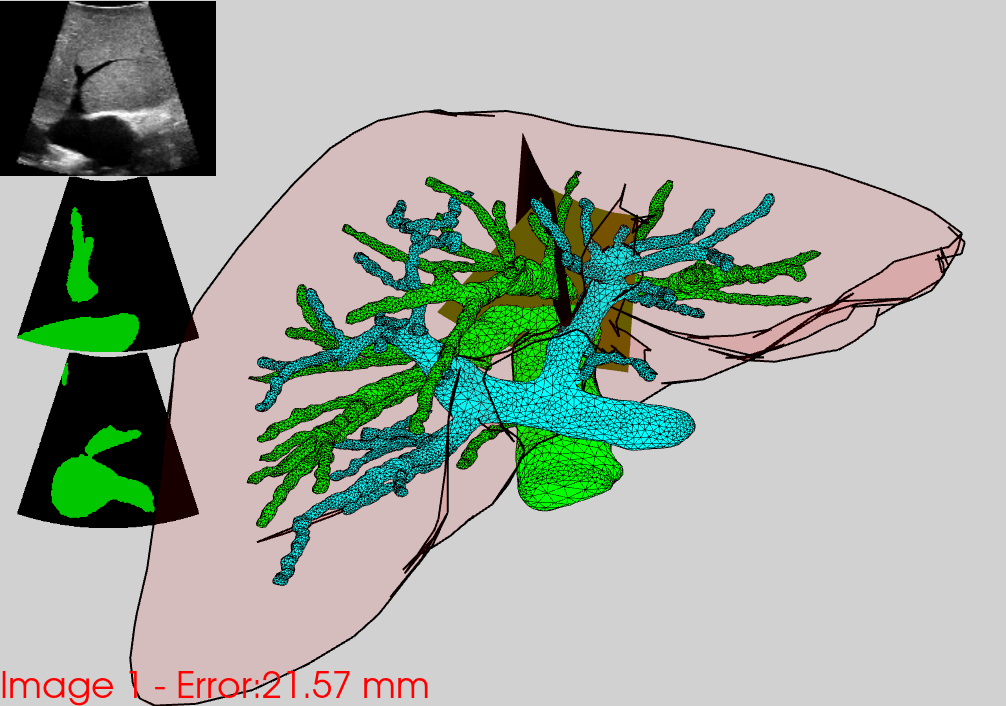

Supplement: Supplementary file 10 — DH result for Sweep 1 of Case 4 (3838 KB) [file 11548_2022_2605_MOESM10_ESM.gif]

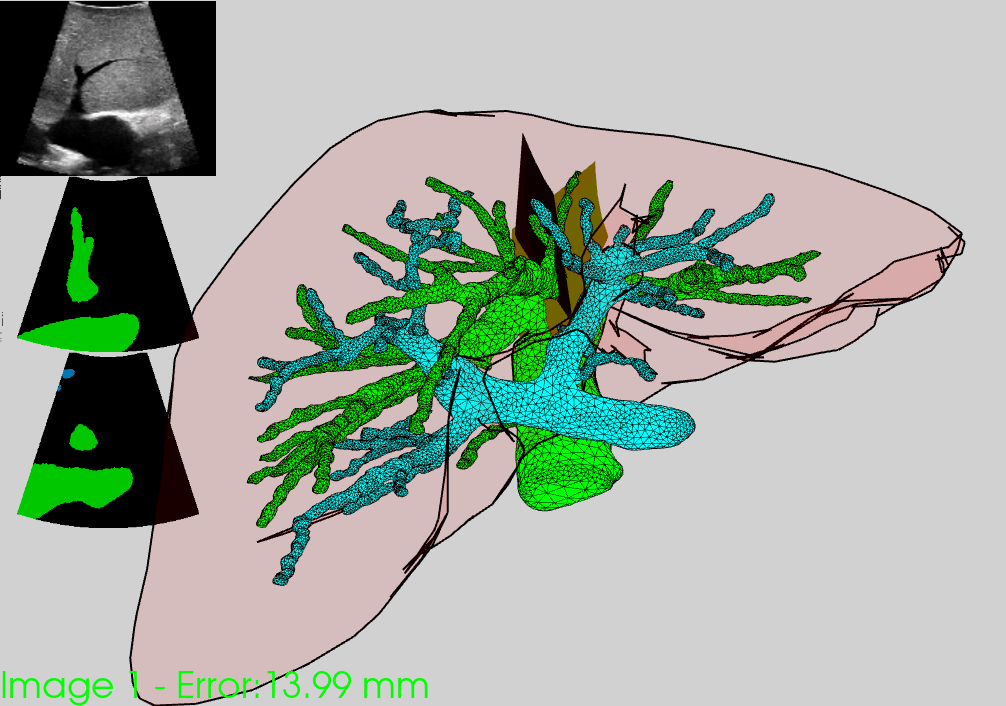

Supplement: Supplementary file 11 — Handcrafted CBIR result for Sweep 1 of Case 4 (3814 KB) [file 11548_2022_2605_MOESM11_ESM.gif]
